# Activation of microglial NADPH oxidase is synergistic with glial iNOS expression in inducing neuronal death: a dual-key mechanism of inflammatory neurodegeneration

**DOI:** 10.1186/1742-2094-2-20

**Published:** 2005-09-12

**Authors:** Palwinder Mander, Guy C Brown

**Affiliations:** 1 Biochemistry Department, University of Cambridge, Tennis Court Road, Cambridge, CB2 1QW, UK

**Keywords:** microglia, peroxynitrite, nitric oxide, prion protein, inflammation, cytokines

## Abstract

**Background:**

Inflammation-activated glia are seen in many CNS pathologies and may kill neurons through the release of cytotoxic mediators, such as nitric oxide from inducible NO synthase (iNOS), and possibly superoxide from NADPH oxidase (NOX). We set out to determine the relative role of these species in inducing neuronal death, and to test the dual-key hypothesis that the production of both species simultaneously is required for significant neuronal death.

**Methods:**

Primary co-cultures of cerebellar granule neurons and glia from rats were used to investigate the effect of NO (from iNOS, following lipopolysaccharide (LPS) and/or cytokine addition) or superoxide/hydrogen peroxide (from NOX, following phorbol 12-myristate 13-acetate (PMA), ATP analogue (BzATP), interleukin-1β (IL-1β) or arachidonic acid (AA) addition) on neuronal survival.

**Results:**

Induction of glial iNOS caused little neuronal death. Similarly, activation of NOX alone resulted in little or no neuronal death. However, if NOX was activated (by PMA or BzATP) in the presence of iNOS (induced by LPS and interferon-γ) then substantial delayed neuronal death occurred over 48 hours, which was prevented by inhibitors of iNOS (1400W), NOX (apocynin) or a peroxynitrite decomposer (FeTPPS). Neurons and glia were also found to stain positive for nitrotyrosine (a putative marker of peroxynitrite) only when both iNOS and NOX were simultaneously active. If NOX was activated by weak stimulators (IL-1β, AA or the fibrillogenic prion peptide PrP106-126) in the presence of iNOS, it caused microglial proliferation and delayed neurodegeneration over 6 days, which was prevented by iNOS or NOX inhibitors, a peroxynitrite decomposer or a NMDA-receptor antagonist (MK-801).

**Conclusion:**

These results suggest a dual-key mechanism, whereby glial iNOS or microglial NOX activation alone is relatively benign, but if activated simultaneously are synergistic in killing neurons, through generating peroxynitrite. This mechanism may mediate inflammatory neurodegeneration in response to cytokines, bacteria, ATP, arachidonate and pathological prions, in which case neurons may be protected by iNOS or NOX inhibitors, or scavengers of NO, superoxide or peroxynitrite.

## Background

Glia (microglia and astrocytes) can become inflammation activated in many CNS pathologies, including infectious, ischaemic, inflammatory and neurodegenerative disorders [1,2]. Glial activation may be protective to the host, as it can lead to the removal of cell debris and killing of pathogens [3]. However excessive or chronic glial activation can kill nearby neurons [4,5]. Thus inflammation may contribute to many CNS pathologies including Alzheimer's, Parkinson's and motor neuron diseases, multiple sclerosis, meningitis, AIDS dementia, strokes, trauma and normal brain ageing [6,7]. It is therefore important to understand the mechanisms by which inflammatory-activated glia kill neurons.

Astrocytes and microglia can become activated by a range of factors, including pathogens and pro-inflammatory cytokines, and can lead to the subsequent death of co-cultured neurons [8,9]. Activated astrocytes and/or microglia produce a variety of factors which can mediate neuronal death, including reactive oxygen species (ROS) [10,11], nitric oxide [8,9,12] and glutamate [8,13], as well as pro-inflammatory cytokines that perpetuate glial activation, such as interleukin-1β (IL-1β) and tumour necrosis factor-α (TNF-α) [14].

The neuroprotective effects of anti-oxidants have been established [15] and are thought to be due to the removal of ROS (such as superoxide) and as well as more toxic molecules (such as peroxynitrite) [16]. There is evidence that NADPH oxidase is activated in Alzheimer's disease and AIDS dementia [17-19]. The major source of ROS during inflammation is NADPH oxidase [20,21], although other sources may also contribute [22,23]. NADPH oxidase is expressed mainly by microglia in the brain [21,24], and produces superoxide (O_2_^-^) extracellularly or within phagocytic vesicles, in order to kill pathogens. The oxidase can be acutely activated by PMA, ATP, arachidonic acid, some chemokines and cytokines [25-28]. Superoxide is then broken down mainly by extracellular and intracellular superoxide dismutase to give hydrogen peroxide (H_2_O_2_).

iNOS is not normally expressed in the brain, but is induced in astrocytes and microglia by proinflammatory cytokines and pathogen components, such as lipopolysaccharide (LPS)/endotoxin of Gram-negative bacteria [29]. Once expressed iNOS produces high, sustained levels of NO which can, in certain conditions, kill nearby neurons, by mechanisms including inhibition of mitochondrial respiration and the release of glutamate from neurons and glia, resulting in excitotoxicity [8]. However, such mechanisms may require a relatively high level of NO and/or a relatively low level of oxygen [30,31]. An alternative mechanism would be for NO to react with superoxide (e.g. from the NADPH oxidase) to produce peroxynitrite (ONOO-), which is potentially more neurotoxic to neurons than NO or superoxide [32,33].

This suggests a dual-key hypothesis of inflammatory neurodegeneration whereby iNOS expression or NADPH oxidase activation alone is relatively benign, but when combined together at the same time causes neurodegeneration via peroxynitrite. We have previously shown that acute activation of the NADPH oxidase in isolated microglia expressing iNOS results in the rapid disappearance of NO and produces ONOO^- ^[32]. In this paper we report that activation of the microglial NADPH oxidase to produce superoxide is synergistic with NO from iNOS in inducing death of co-cultured neurons, whereas activation of either alone causes little or no death of co-cultured neurons.

## Materials & methods

### Materials

The following materials were purchased from the indicated sources: 1400W.dihydrochloride from Alexis (Nottingham, UK); MK-801 maleate, apocynin and FeTPPS (5,10,15,20-Tetrakis(4-sulfonatophenyl)porphyrinato Iron (III) chloride) from Calbiochem (Nottingham, UK). All other reagents were ordered from Sigma (Poole, UK).

### Neuronal-glial culture

Cerebellar granule cell (CGC) cultures were prepared from 7-day-old Wistar rats, as described in Bal-Price & Brown, 2001. Briefly, the pups were anaesthetised using 5% halothane in oxygen, followed by decapitation. Brains were removed under sterile conditions and the cerebellum dissected. Meninges were removed and the cerebella dissociated in Versene solution (1:5000, Gibco BRL) and plated at 0.25 × 10^6 ^cells/cm^2 ^in 24-well plates (in 500 μl DMEM) coated with 0.001% poly-L-lysine. Cultures were maintained in DMEM (Gibco BRL) supplemented with 5% horse serum, 5% foetal calf serum, 38 mM glucose, 5 mM HEPES, 2 mM glutamine, 25 mM KCl and 10 μg/ml gentamicin. Cells were kept at 37°C in a humidified atmosphere of 5% CO_2_/95% air and used for experiments at 16–18 days *in vitro *(DIV). Cultures of CGC's contained 22 ± 4% astrocytes and 2 ± 1% microglia as assessed by immunocytochemistry using antibodies against glial fibrillary acidic protein (GFAP: a marker for astrocytes) and complement receptor-3 (a marker for microglia), CGC's were identified based on morphology and at 16–18 DIV 76 ± 5% of the cells in the culture were CGC's. All experiments were undertaken in accordance with the UK Animals (Scientific Procedures) Act 1986.

### Activation of glia in neuronal-glial cultures

Lipopolysaccharide (LPS), a cell wall component of Gram-negative bacteria and interferon-γ (IFN-γ), a pro-inflammatory cytokine, are potent activators of glia when administered together. Neuronal-glial cultures were treated with 100 ng/ml LPS (from *Salmonella typhimurium*) and 10 ng/ml IFN-γ (rat recombinant, Sigma) for 48 hours (or longer where indicated). The proinflammatory cytokines tumour necrosis factor-α (TNF-α; 10 ng/ml, rat recombinant, Sigma) and interleukin-1β (IL-1β; 10 ng/ml, rat recombinant, Sigma) were also used in combination with IFN-γ to activate the glia in neuronal-glial cultures (48 hours). Where present, inhibitors were added at the same time as LPS/IFN-γ.

In some experiments IL-1β or arachidonic acid (AA, 30 μM) were added to the cultures as well as LPS/IFN-γ. In these experiments, IL-1β or AA were added 24 hours after LPS/IFN-γ addition, but inhibitors were added at the same time as LPS/IFN-γ. Activators, inhibitors and IL-1β or AA were added once only and neuronal death was assessed 144 hours after LPS/IFN-γ addition.

In some experiments, prion protein or a fragment of the human prion protein were used (kindly provided by David R. Brown, University of Bath). Recombinant mouse prion protein was expressed in bacteria and purified using a histidine-tagging method, as described previously [34]. The prion peptide (PrP106-126) with sequence KTNMKHMAGAAAAGAVVGGLG was derived from amino acid residues 106–126 of the human prion protein sequence, and a scrambled sequence of the peptide was used as a control; sequence: NGAKALMGGHGATKVMVGAAA. Prion protein was used at 5 μg/ml and the prion protein peptides at 225 μg/ml.

To activate NADPH oxidase, phorbol 12-myristate 13-acetate (PMA, 50 ng/ml) or benzoyl(benzoyl)-ATP (BzATP, 1 mM) are used and are added to neuronal-glial cultures either alone or at the same time as LPS/IFN-γ.

### Enrichment of microglia in neuronal-glial cultures

Primary rat microglia were obtained from mixed glial cultures (astrocytes and microglia). Glial cultures were prepared from the cerebral cortices of 7-day-old Wistar rats (the same brains that were used to isolate cerebellar granule neurons). Meninges were removed from the cerebral hemispheres and then dissociated using a solution of EBSS containing 0.3% BSA, 0.004% DNase I and 0.025% Trypsin. Cells were plated at 0.1 × 10^6 ^cells/cm^2 ^in 75 cm^2 ^cell culture flasks (Falcon) coated with 0.0005% poly-L-lysine. Cultures were maintained in DMEM supplemented with 10% foetal calf serum and 1% Penicillin-Streptomycin. Cells were kept at 37°C in a humidified atmosphere of 5% CO_2_/95% air.

At confluency, glial cultures were used to isolate microglial cells by gently shaking/tapping the mixed glial cultures to dislodge microglia loosely attached to astrocytes. Medium from the mixed glial cultures, containing microglia was removed and centrifuged (135 g for 5 minutes). Microglia were re-suspended in conditioned medium from CGC cultures and added to neuronal-glial cultures in some experiments (50, 000 microglia/cm^2^). Fifteen minutes after the addition of microglia to some neuronal-glial cultures, LPS/IFN-γ and inhibitors where appropriate were added together. Neuronal death was assessed 48 hours after LPS/IFN-γ addition.

### Assessment of glial activation

Activation of glia in the neuronal-glial culture was assessed by NADPH diaphorase staining and measurements of nitrite in the medium. Nitric oxide synthase (NOS) is an NADPH diaphorase, using a chromogen (nitroblue tetrazolium, NBT), and NADPH as the reductant, diaphorase staining was used to detect cells with NOS activity. Following treatment (with cytokines or untreated for control staining) the neuronal glial cultures were fixed with 4% paraformaldehyde in phosphate buffer for 30 minutes at 4°C. After fixation, cells were incubated in 0.3% Triton X-100 (in phosphate buffer) for 5 minutes. Cells were then incubated for 2 hours at 37°C in 0.3% Triton X-100 containing 1 mg/ml NADPH and 0.2 mg/ml NBT. Cells were washed once with 0.3% Triton X-100 and then viewed using an inverted light microscope (Leica).

Nitrite levels in the medium were measured using the Griess reaction. Briefly, aliquots of medium following treatments were taken and centrifuged (8000 *g *for 5 minutes). 6 mM HCl was added to the supernatant and then 1 mM sulfanilamide and 1 mM N-1 (1-naphthyl)ethylenediamine (NEDA) were added. Absorbance at a wavelength of 548 nm was measured by plate reader (BMG, Fluostar Optima), before and after the addition of NEDA. Nitrite concentrations in samples were calculated from a standard curve of sodium nitrite in DMEM.

### Assessment of cell viability

The viability of CGC's was assessed by propidium iodide (PI, 2 μg/ml) and Hoechst 33342 (6 μg/ml) staining, using a fluorescence microscope (Axiovert S-100) and filters for excitation at 365 nm and emission at 420 nm. The cell-impermeable nuclear dye, PI stains the nuclei of cells that have lost plasma membrane integrity and are considered to be necrotic. Using the cell-permeable DNA dye Hoechst 33342, the nuclear morphology of the CGC's was studied. Neuronal nuclei exhibiting irregular Hoechst staining, nuclear shrinkage, chromatin condensation and/or nuclear fragmentation but PI negative were classified as showing chromatin condensation (CC). Individual cells exhibiting both CC and PI staining were included in the PI data. Cells were counted in three microscopic fields in each well (3 wells per treatment) and expressed as a percentage of the total number of neurons. Each treatment was repeated at least three times.

### Assessment of microglia proliferation

Microglia cells were identified using Isolectin IB_4 _(from *Griffonia simplicifolia*), which has strong affinity for microglia but not astrocytes. An Alexa Fluor 488 conjugate of isolectin IB_4 _(10 ng/ml) was added to cultures activated with LPS/IFN-γ and treated with IL-1β, AA or prion protein/peptide and incubated for 15 minutes at 37°C. Stained cells (microglia) were visualised and counted by viewing under a fluorescence microscope (excitation 488 nm, emission 530 nm).

### 3-nitrotyrosine immunocytochemistry

Mixed neuronal-glial cultures were stained for the peroxynitrite marker, 3-nitrotyrosine (3-NT). Cultures were untreated (control) or treated with LPS/IFN-γ, PMA, LPS/IFN-γ/PMA or FeTPPS + LPS/IFN-γ/PMA. Cultures were fixed with 4% paraformaldehyde and then incubated with 10 μg/ml of anti-nitrotyrosine monoclonal antibody (Upstate). The primary antibody was detected using a Cy3-conjugated secondary antibody (Jackson ImmunoResearch Laboratories). 3-NT -positive cells were visualised using a fluorescence microscope (excitation 546 nm, emission 590 nm).

### Statistical analysis

Data are expressed as mean ± SEM and were analysed for significance using ANOVA.

## Results

### Inflammatory activation of glia in neuronal-glial cultures does not lead to substantial death of the co-cultured neurons

A mature mixed culture (16–18 days in vitro) of cerebellar granule neurons and glia (22% astrocytes and 2% microglia) was used to investigate inflammation-activated glia-induced neuronal death. The glia in the neuronal-glial cultures were activated with a combination of endotoxin (lipopolysaccharide, LPS) and a pro-inflammatory cytokine (interferon-γ, IFN-γ) or different combinations of pro-inflammatory cytokines including tumour necrosis factor-α (TNF-α) and interleukin-1β (IL-1β). Neuronal death was assessed 48 hours after treatment with the inflammatory activators (LPS/IFN-γ, TNF-α/IFN-γ, IL-1β/IFN-γ or TNF-α/IL-1β/IFN-γ). Two nuclear dyes were used to stain the cultures and assess for necrosis and apoptosis: cell-impermeable propidium iodide (PI) to stain necrotic cells and the cell-permeable Hoechst 33342 used to characterise any neuronal nuclei showing signs of chromatin condensation or nuclear fragmentation (characteristic of apoptosis). Although relatively small, significant levels of neuronal death were induced following activation with LPS/IFN-γ, TNF-α/IFN-γ or TNF-α/IL-1β/IFN-γ but not IL-1β/IFN-γ (Table 1).

**Table 1:** Effects of inflammatory activated-glia in mixed neuronal-glial cultures on neuronal death. Neuronal death was assessed by propidium iodide staining (PI, necrosis) or chromatin condensation of neuronal nuclei by Hoechst 33342 staining (CC, a marker of apoptosis) 48 hours after treatment. Nitrite (the primary breakdown product of NO) levels were measured in the culture medium 48 hours following treatments. Statistical differences were established using ANOVA at *p < 0.05 and ***p < 0.001. Data expressed is mean ± SEM, n = 3 or more.

**TREATMENT**	**PI (%)**	**CC (%)**	**NITRITE (μM)**
UNTREATED	0.9 ± 0.9	0.5 ± 0.6	2.7 ± 3.0
LPS/IFN-γ	5.7 ± 3.4 *	3.6 ± 1.5 *	18.6 ± 8.4 ***
TNF-α/IFN-γ	5.6 ± 0.4 ***	4.3 ± 2.9 *	4.2 ± 2.8
IL-1β/IFN-γ	1.1 ± 1.1	0.6 ± 0.7	3.7 ± 2.1
TNF-α/IL-1β/IFN-γ	6.3 ± 4.1 *	5.5 ± 3.2 *	4.5 ± 1.4

**Figure 1:**
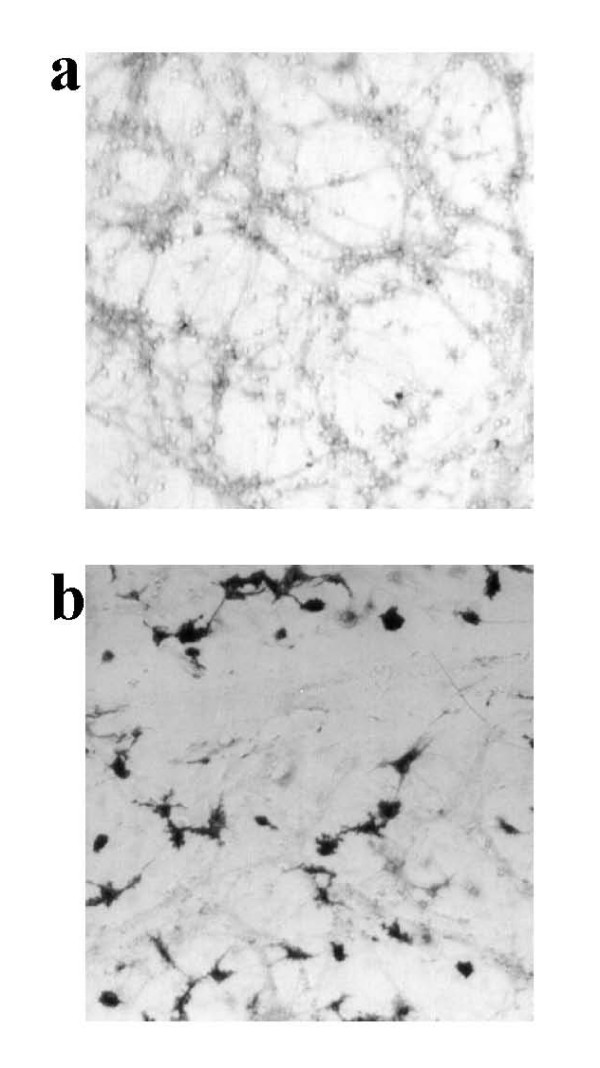
**NOS activity in mature neuronal-glial cultures. **NADPH diaphorase staining was used to assess for NOS activity. Non-activated (control) cultures show weak staining in neurons and along their processes (a), but following LPS/IFN-γ treatment (b) dark staining is visible in glia (astrocytes and microglia). The photographs shown are representative and were taken 48 hours after treatment, n>3.

To confirm that the glia in the culture had actually been activated to express iNOS, we used a simple stain for nitric oxide synthase (NOS) activity (NADPH diaphorase staining) enabling us to visualise cells with NOS activity and distinguish between microglia and astrocytes based on morphology. Additionally we assessed nitrite levels in the culture medium as a measure of NO production (Table: 1). Non-activated cultures showed no NADPH diaphorase staining in glia, but low-level staining was seen in neurons (probably due to nNOS) and correlates with the low level of nitrite present in the medium (Figure: 1a; Table: 1). However, after treatment with LPS/IFN-γ, a high proportion of glia (both microglia and astrocytes) stained intensely for diaphorase activity (Figure: 1b). Treatment with TNF-α/IFN-γ, IL-1β/IFN-γ or TNF-α/IL-1β/IFN-γ resulted in much less diaphorase staining of glia, and little or no nitrite elevation, indicating a requirement for LPS to induce substantial iNOS expression.

Relatively pure neuronal cultures (CGC cultures isolated as described in the methods section and then treated with 10 μM arabinoside cytosine at 18 hours to inhibit the proliferation of glia) consisting of 97 ± 4% neurons, 2 ± 1% astrocytes and 1 ± 1% microglia were not affected by the presence of cytokines alone (mean % of chromatin-condensed (CC) and propidium iodide-positive (PI) neurons ± SEM of 3 separate cultures; control: CC: 4 ± 3%, PI: 8 ± 4%; 10 ng/ml IL-1β: CC: 3 ± 2%, PI: 7 ± 3%; 10 ng/ml TNF-α: CC: 3 ± 2%, PI: 9 ± 4%). Additionally, no significant adverse effects were seen even if the concentrations of IL-1β or TNF-α were increased 10-fold (mean % of neurons ± SEM of 1 culture; control: CC: 4 ± 3%, PI: 8 ± 4%; 100 ng/ml IL-1β: CC: 4 ± 2%, PI: 5 ± 4%; 100 ng/ml TNF-α: CC: 4 ± 2%, PI: 6 ± 4%) or if combined with 10 ng/ml IFN-γ treatment (mean % of neurons ± SEM of 2 separate cultures; control: CC: 4 ± 3%, PI: 8 ± 4%; 10 ng/ml IL-1β + IFN-γ: CC: 3 ± 2%, PI: 5 ± 3%; 10 ng/ml TNF-α + IFN-γ: CC: 3 ± 3%, PI: 7 ± 2%).

These results suggest that the cytokines have no direct toxicity for neurons, and although nitric oxide (NO) is produced by iNOS expressed in glia following activation with LPS/IFN-γ, it is not able to kill the co-cultured neurons alone, or the quantities of NO produced are not sufficient to induce widespread death of these mature neuronal cultures.

### Simultaneous activation of iNOS and NADPH oxidase results in massive neuronal death, mediated by peroxynitrite

**Figure 2:**
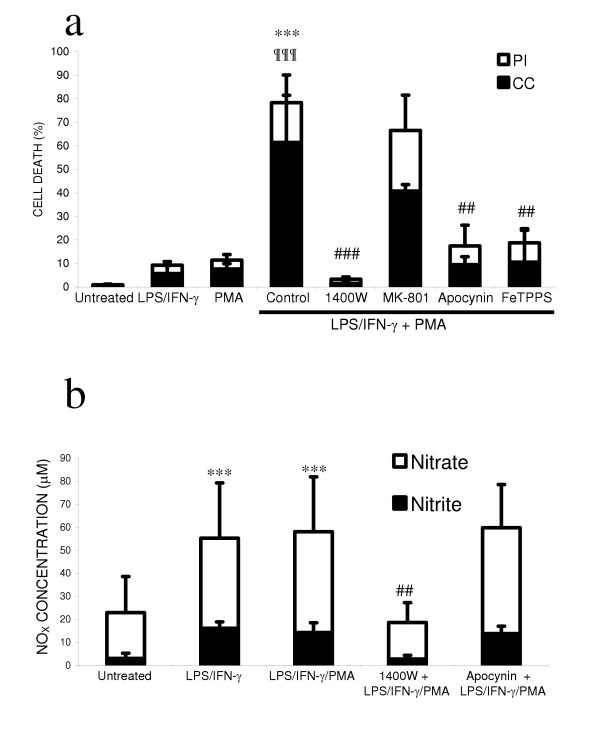
**Activation of NADPH oxidase in the presence of glial iNOS is synergistic in killing co-cultured neurons. **Cultures were stained with the cell-impermeable dye propidium iodide (PI) to count necrotic cells and the cell-permeable dye Hoechst 33342 to count neuronal nuclei showing chromatin condensation/fragmentation (CC), 48 hours after treatment (a). PMA stimulation of NADPH oxidase did not substantially affect neuronal survival, but in the presence of LPS/IFN-γ had synergistic effects on neuronal death, which were blocked by inhibitors of iNOS (25 μM 1400W), NADPH oxidase (1 mM apocynin), or a peroxynitrite scavenger (100 μM FeTPPS) but not by a blocker of the NMDA receptor (10 μM MK-801). Nitrite and nitrate levels were not affected by the presence of PMA or apocynin but were significantly reduced by 1400W (b). Statistical differences were established using ANOVA at *p <0.05, **p < 0.01 and ***p < 0.001, the symbol # replaces * when comparing protection against LPS/IFN-γ/PMA induced neuronal death. The symbol ¶ is used to demonstrate a significant difference in comparison to PMA or LPS/IFN-γ alone. Statistical significance refers to the total death (black + white parts of the bar). Data expressed is mean ± SEM, n = 3 or more.

As NO produced by inflammatory activated glia did not induce substantial neuronal death, we investigated whether simultaneous production of superoxide resulting in peroxynitrite would be more toxic to neurons. Peroxynitrite is formed from the diffusion-limited reaction of NO with superoxide. Under inflammatory conditions in the brain, NADPH oxidase is the major source of superoxide, therefore we used phorbol 12-myristate 13-acetate (PMA) to activate this enzyme and generate a source of superoxide in the neuronal-glial culture. As the number of NADPH diaphorase-positive glia was greatest following treatment with LPS/IFN-γ, we used LPS/IFN-γ to induce iNOS expression in the glia and provide a source of NO. We found that treating neuronal-glial cultures with LPS/IFN-γ/PMA for 48 hours induced extensive neuronal death (Figure: 2a). Treatment of the cultures with PMA alone induced only low levels of neuronal death, similar to that seen with LPS/IFN-γ treatment alone. However, activation of both NADPH oxidase and iNOS was synergistic in inducing neuronal death. This neuronal death was prevented by an iNOS inhibitor of  (1400W), a NADPH oxidase (apocynin) a peroxynitrite scavenger (FeTPPS), but not by a blocker of the NMDA receptor (MK-801).

As PMA activates the protein kinase C pathway, the effects of PMA might be due to reasons other than stimulating the microglial NADPH oxidase, such as increased iNOS expression leading to more NO production and neuronal death by NO and not peroxynitrite. However, the levels of nitrite and nitrate in the culture medium of neuronal-glial cultures treated with LPS/IFN-γ/PMA were not different to those found in the absence of PMA (Figure: 2b).

**Figure 3:**
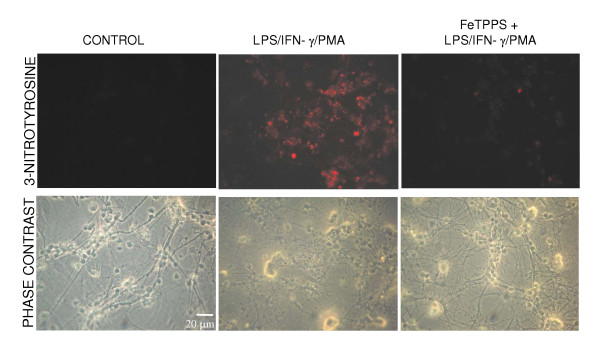
**Activation of NADPH oxidase in the presence of iNOS expression leads to 3-nitrotyrosine immunoreactivity in neurons and glia. **Neuronal-glial cultures treated with LPS/IFN-γ/PMA for 48 hours showed extensive immunoreactivity for 3-nitrotyrosine, which was absent in the presence of FeTPPS. Untreated cultures (control) showed no staining for 3-nitrotyrosine. The photographs shown are representative and were taken 48 hours after treatment, n>3.

To determine whether peroxynitrite generated by glia reaches the neurons, the neuronal-glial cultures were tested for nitrotyrosine immunoreactivity. Positively stained neurons (and glia) were only seen following treatment with LPS/IFN-γ/PMA (Figure: 3) and not in the presence of the peroxynitrite scavenger FeTPPS or when treated with LPS/IFN-γ (data not shown) or PMA alone (data not shown). However, no PI-positive glia or changes in glial nuclear morphology were observed in any of the conditions, implying that although they were exposed to peroxynitrite it did not induce glial death.

**Figure 4:**
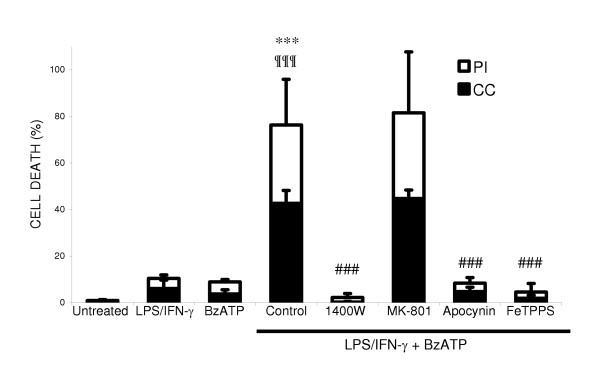
**NADPH oxidase stimulation by P2X7 receptor activation in the presence of glial iNOS kills co-cultured neurons. **Neuronal death was assessed by propidium iodide staining (PI) and chromatin condensation of neuronal nuclei by Hoechst 33342 staining (CC) 48 hours after treatment. Neuronal death induced by BzATP following LPS/IFN-γ activation, was prevented by inhibitors of iNOS (25 μM 1400W), NADPH oxidase (1 mM apocynin) and a peroxynitrite scavenger (100 μM FeTPPS) but not by a blocker of the NMDA receptor (10 μM MK-801). Statistical differences were established using ANOVA at *p < 0.05 and ***p < 0.001, the symbol # replaces * when comparing protection against LPS/IFN-γ/BzATP induced neuronal death. Statistical significance refers to the total death (black + white parts of the bar). Data expressed is mean ± SEM, n = 3 or more.

ATP is known to be released by neurons and glia in a variety of conditions, and has been reported to activate the microglial NADPH oxidase via P2X7 receptors [26]. We found that ATP rapidly stimulated superoxide/hydrogen peroxide production by isolated microglia, which was sensitive to diphenyleneiodonium (DPI), an inhibitor of NADPH oxidase (ATP: 80 ± 7 picomoles H_2_O_2_/minute/1 × 10^5 ^microglia). However, ATP did not induce neuronal death alone, or in synergy with LPS/IFN-γ treatment (data not shown), probably because it is rapidly hydrolysed in cell culture medium [35]. Therefore, we used a non-hydrolysable ATP analogue, 2'-3'-O-(4- benzoylbenzoyl)-ATP (BzATP), known to be a specific P2X7 receptor agonist [36]. BzATP was also found to stimulate DPI-sensitive hydrogen peroxide production by isolated microglia, which was comparable to that produced by PMA (control: 12 ± 3; PMA: 204 ± 50; BzATP: 124 ± 15 picomoles H_2_O_2_/minute/1 × 10^5 ^microglia). BzATP did not induce neuronal death alone but had synergistic effects on neuronal death in the presence iNOS expression (Figure: 4). LPS/IFN-γ/BzATP induced neuronal death was blocked by inhibitors of iNOS, NADPH oxidase and a peroxynitrite scavenger, but not by the NMDA receptor blocker.

### Activation of glia in microglia-enriched neuronal-glial cultures potently kills co-cultured neurons

**Figure 5:**
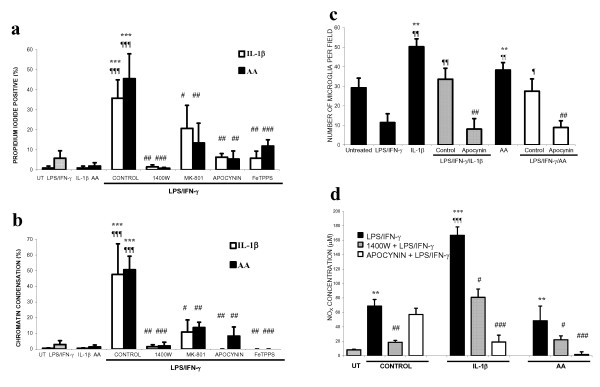
**Effects of IL-1β or arachidonic acid (AA) on neuronal survival in the presence of inflammation-activated glia in neuronal-glial cultures. **Neuronal death was assessed by propidium iodide staining (PI; a) or chromatin condensation of neuronal nuclei (CC; b) after 6 days of treatment. Neuronal death was prevented by inhibitors of iNOS (25 μM 1400W), NADPH oxidase (1 mM apocynin), a blocker of the NMDA-receptor (10 μM MK-801) or a peroxynitrite scavenger (100 μM FeTPPS). Neuronal death was accompanied by proliferation of microglia (c). Microglial proliferation was inhibited by LPS/IFN-γ treatment alone but in the presence of IL-1β or AA it was stimulated and returned to basal levels. This stimulation of proliferation by IL-1β or AA (in the presence of LPS/IFN-γ) was completely prevented by apocynin. Additionally, nitrite/nitrate (NO_X_) levels correlated with the number of microglia present (d). Statistical differences were established using ANOVA at *p < 0.05, **p < 0.01 and ***p < 0.001, the symbol * is used when assessing prevention of neuronal death in comparison to LPS/IFN-γ with IL-1β or AA. The symbol ¶ is used when comparing neuronal death to that induced by LPS/IFN-γ alone and # when comparing neuronal death induced by IL-1β or AA treatment alone. In c & d, the differences are in comparison to IL-1β or AA alone (*), LPS/IFN-γ (¶) or LPS/IFN-γ plus IL-1β or AA (#). Data expressed is mean ± SEM, n = 3 or more.

We have found that IL-1β or arachidonic acid (AA) can activate the microglial NADPH oxidase, although to lesser extent than PMA (control: 12 ± 3; IL-1β: 37 ± 20; AA: 24 ± 4 picomoles H_2_O_2_/minute/1 × 10^5 ^microglia). We therefore tested whether IL-1β or AA could synergise with LPS/IFN-γ to induce neuronal death. The addition of either IL-1β or AA did not induce further neuronal death than that induced by LPS/IFN-γ alone up to 48 hours after additions (data not shown). However if such cultures were maintained for 6 days, we found that widespread neuronal death occurred (Figure: 5a, b) and was blocked by inhibitors of iNOS, NADPH oxidase, a peroxynitrite scavenger and a blocker of the NMDA receptor. Treatment with IL-1β or AA alone did not have any effect on neuronal survival, but did increase the number of microglia in neuronal-glial cultures (Figure: 5c). Treatment with LPS/IFN-γ was found to inhibit microglia proliferation but in the presence of IL-1β or AA this inhibition was overcome and lead to a progressive increase in the number of microglia and subsequent neuronal death. The mitogenic effects of IL-1β or AA are probably mediated by hydrogen peroxide following stimulation of NADPH oxidase (unpublished data) and we found that the NADPH oxidase inhibitor, apocynin, prevented this increase in the number of microglia. Nitrite and nitrate (NO_X_) levels (Figure: 5d) were higher in cultures treated with IL-1β or AA plus LPS/IFN-γ, but not in the presence of apocynin, which blocked microglial proliferation, suggesting that microglia were the predominant source of NO and/or peroxynitrite.

**Figure 6:**
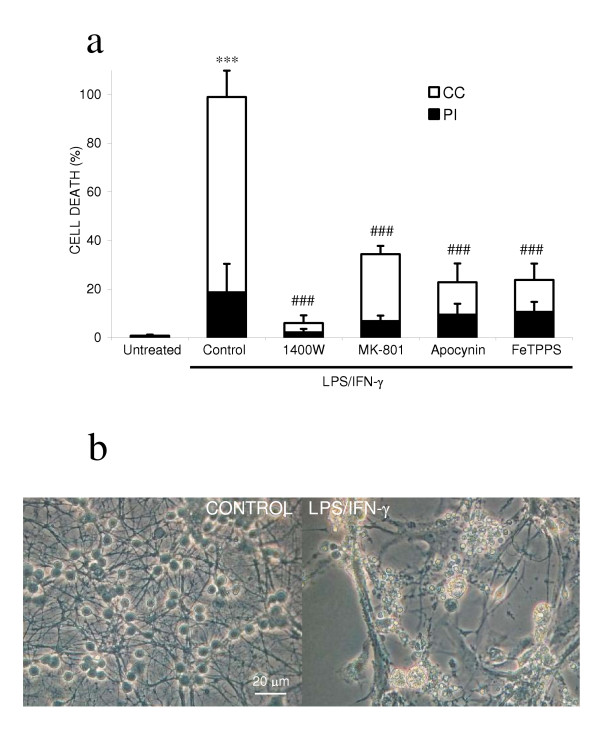
**Activation of microglia-enriched neuronal-glial cultures induces complete neurodegeneration. **The microglia population was enriched in neuronal-glial cultures by adding isolated microglia (50,000 microglia/cm^2^). Neuronal death was assessed by propidium iodide staining (PI) or chromatin condensation of neuronal nuclei (CC) at 48 hours after treatment (a). Neuronal death was prevented by inhibitors of iNOS (25 μM 1400W), NADPH oxidase (1 mM apocynin), a blocker of the NMDA-receptor (10 μM MK-801), or a peroxynitrite scavenger (100 μM FeTPPS). LPS/IFN-γ activation of the microglia-enriched neuronal-glial cultures led to complete disintegration of neuronal processes and severe shrinkage of neuronal cell bodies (b). Statistical differences were established using ANOVA at *p < 0.05 and ***p < 0.001, in comparison to control (added microglia) non-activated cultures, and the symbol # replaces * when comparing protection against neuronal death induced by LPS/IFN-γ activated cultures. Statistical significance refers to the total death (black + white parts of the bar). Data expressed is mean ± SEM, n = 3 or more. Photographs shown are representative and were taken 48 hours after the addition of LPS/IFN-γ.

Since IL-1β and AA stimulated microglial proliferation (in the presence or absence of LPS/IFN-γ), we wanted to test whether increasing the microglial density would sensitise to LPS/IFN-γ induced neuronal death. So we investigated whether enriching the microglia population in the neuronal-glial culture followed by inflammatory activation would result in widespread neuronal death. The neuronal-glial culture used in the last section was enriched with microglia by adding freshly isolated microglia. LPS/IFN-γ activation of a microglia-rich (15% microglia as opposed to 2%) neuronal-glial culture resulted in all neurons rapidly losing their dendritic processes and shrinkage of the cell body (Figure: 6b), in addition to chromatin condensation or propidium iodide staining of the nuclei at 48 hours of treatment (Figure: 6a). This neuronal death was prevented by inhibitors of iNOS, NADPH oxidase, a peroxynitrite decomposition catalyst and a blocker of the NMDA receptor. The addition of microglia alone (non-activated) did not affect neuronal survival (Figure: 6a, untreated). In support of microglia as the key cell type in inflammatory neurodegeneration, LPS/IFN-γ activation of astrocyte-enriched neuronal-glial cultures did not lead to widespread killing of co-cultured neurons (isolation of neuronal-glial cultures as normal but plated onto a confluent bed of astrocytes; data not shown).

### Prion protein or PrP106-126 induce neuronal death in the presence of inflammatory activation mediated by microglia and NADPH oxidase activation

**Figure 7:**
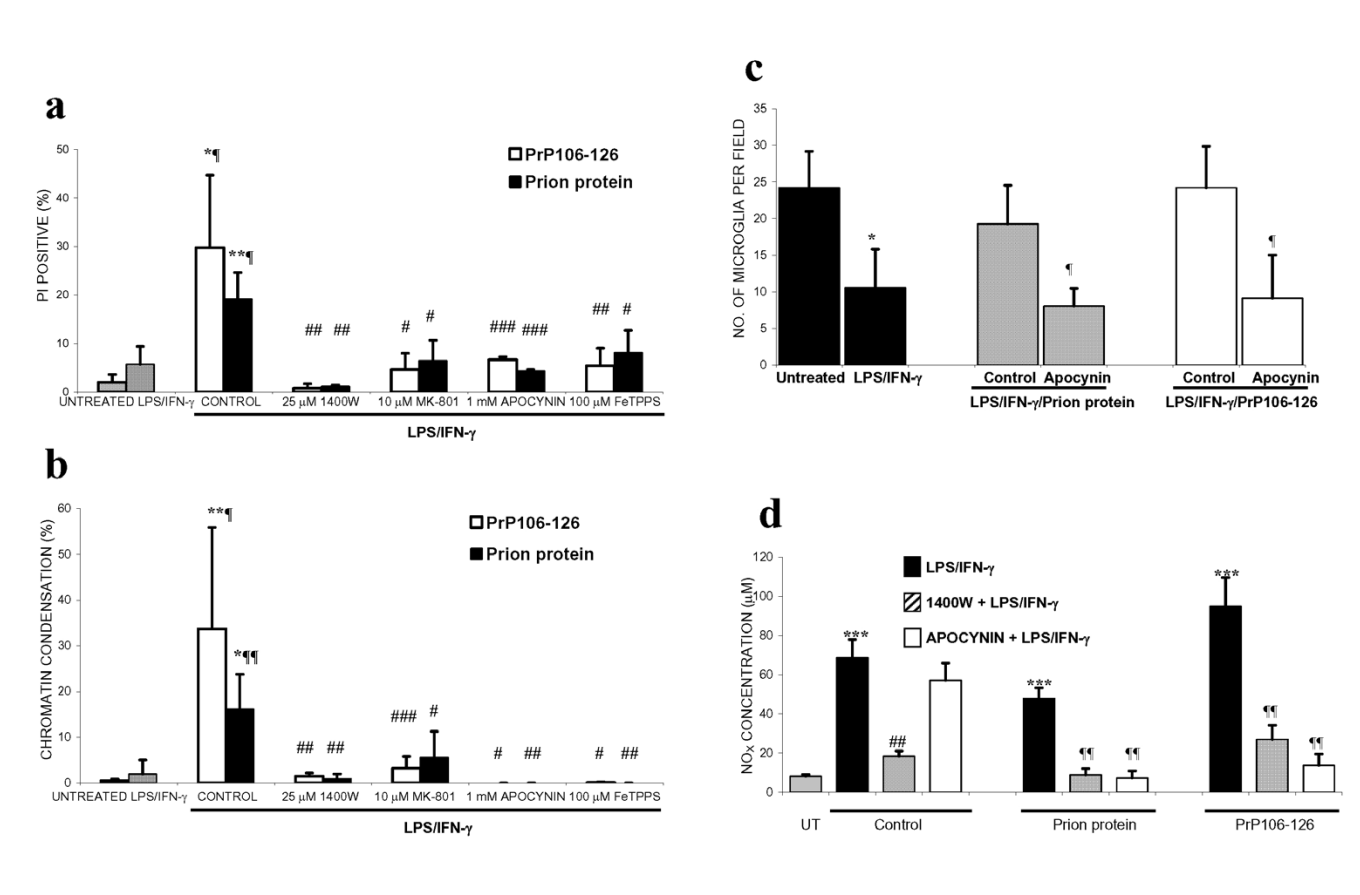
**Delayed neurodegeneration induced by prion protein or PrP106-126 in the presence of iNOS expression is microglia-dependent and mediated by peroxynitrite. **The addition of prion protein (5 μg/ml) or PrP106-126 (225 μg/ml) to LPS/IFN-γ treated neuronal-glial cultures induced delayed death of co-cultured neurons, over 6 days. Neuronal death, assessed by Hoechst 33342 to visualise chromatin condensation (CC; b) or PI for necrosis (a) was prevented by inhibitors of iNOS (25 μM 1400W) and NADPH oxidase (1 mM apocynin), a peroxynitrite scavenger (100 μM FeTPPS) or a blocker of the NMDA receptor (10 μM MK-801). Neuronal death was accompanied by proliferation of microglia (c). Microglial proliferation was inhibited by LPS/IFN-γ treatment alone but in the presence of prion protein or PrP106-126 it was stimulated and returned to basal levels. This stimulation of proliferation by prion protein or PrP106-126 (in the presence of LPS/IFN-γ) was completely prevented by apocynin. Additionally, nitrite/nitrate (NO_X_) levels correlated with the number of microglia present (d). Statistical differences were established using ANOVA at *p < 0.05, **p < 0.01 and ***p < 0.001 are in comparison to untreated cultures (symbol *) or LPS/IFN-γ treatment (symbol ¶) or LPS/IFN-γ plus prion protein or PrP106-126 (symbol #); data expressed is mean ± SEM, n = 3 or more. In c & d, the differences are in comparison to prion protein or PrP106-126 alone (*), LPS/IFN-γ (¶) or LPS/IFN-γ plus prion protein or PrP106-126 (#). Data expressed is mean ± SEM, n = 3 or more.

The prion peptide, PrP106-126, has previously been shown to activate microglia, causing proliferation and ROS production [37,38]. We have recently found that the prion protein and peptide stimulate the NADPH oxidase in isolated microglia (control: 12 ± 3; prion protein: 29 ± 3; PrP106-126: 38 ± 13 picomoles H_2_O_2_/minute/1 × 10^5 ^microglia). We decided to investigate whether the addition of PrP106-126 to iNOS-expressing glia in neuronal-glial cultures would also lead to delayed neurodegeneration, mediated by peroxynitrite and microglia. The addition of prion protein or PrP106-126 alone did not affect neuronal survival in these mature neuronal-glial cultures, but did lead to microglial proliferation (Table: 2). In the presence of glial iNOS (following LPS/IFN-γ treatment), PrP106-126 or prion protein did not exacerbate neuronal death over a period of 2 days, but were synergistic in killing the co-cultured neurons at 6 days (Figure: 7a, b), while a scrambled peptide of the PrP106-126 sequence had no effect. Neuronal death was prevented by blocking NO production from iNOS (1400W), or superoxide from NADPH oxidase (apocynin), through the removal of peroxynitrite (FeTPPS), or by inhibiting the NMDA receptor (MK-801). Additionally, neuronal death was accompanied by microglia proliferation, which was blocked by apocynin (Figure: 7c). Nitrite/nitrate levels were also suppressed in the presence of apocynin, as well as 1400W (Figure: 7d).

**Table 2:** Prion protein or peptide (PrP106-126) does not affect neuronal survival. Neuronal-glial cultures treated once with either prion protein (5 μg/ml) or PrP106-126 (225 μg/ml) did not induce neuronal death over a period of 7 days (assessed by Hoechst 33342 to visualise chromatin condensation (CC) or propidium iodide (PI) to stain necrotic cells). However, prion protein or PrP106-126 did stimulate the proliferation of microglia in neuronal-glial cultures over the same period of time. Statistical differences were established using ANOVA at *p < 0.05, **p < 0.01 and ***p < 0.001 and are in comparison to untreated cultures (symbol *); data expressed is mean ± SEM, n = 3 or more.

**Treatment**	**PI (%)**	**CC (%)**	**Microglia per field**
**Untreated**	2.0 ± 1.6	0.6 ± 0.3	22 ± 5
**Prion protein**	2.9 ± 0.5	0.7 ± 0.6	53 ± 8 ***
**PrP106-126**	1.0 ± 0.8	0.7 ± 0.4	51 ± 7 ***

## Discussion

We found that LPS/IFN-γ induced NOS activity within cultured glia, but induced relatively little death of co-cultured neurons. It has previously been reported that LPS/cytokine-induced iNOS expression in glia is sufficient [5,8,39,40] or insufficient [41-43] to induce death of co-cultured neurons. Similarly, in vivo it has been reported that iNOS expression is sufficient [44,45] or insufficient [46-48] to induce neuronal death. This suggests that either there is a threshold level for NO/iNOS induced neuronal death [49], or NO/iNOS-induced neuronal death is conditional upon some other factors. We have recently reported one such conditional factor (hypoxia) that synergises with NO/iNOS to induce neuronal death [31]. In this report we have tested the hypothesis that NO/iNOS induced neuronal death is conditional upon microglial NADPH oxidase activation.

It has previously been shown that PMA stimulation of microglia results in superoxide production through stimulation of NADPH oxidase [50] and, in the presence of LPS/IFN-γ activated glia (producing NO from iNOS), the superoxide combines with NO to form peroxynitrite [32]. We found that if the NADPH oxidase was stimulated by PMA in the presence of LPS/IFN-γ activated glia, it resulted in extensive death of the co-cultured neurons, while PMA alone induced very little neuronal death. In pathophysiological conditions, extracellular levels of ATP can increase [51], and ATP can activate purinergic receptors (more specifically P2X7 receptors), which can lead to the activation of NADPH oxidase [26]. We used a specific P2X7 receptor agonist (BzATP) to activate the NADPH oxidase in the presence of iNOS expression (LPS/IFN-γ activated cultures) and we found extensive neuronal death, comparable to that induced by LPS/IFN-γ/PMA. Neuronal-glial cultures activated with LPS/IFN-γ/PMA or LPS/IFN-γ/BzATP induced delayed neuronal death that occurred over 2 days. This is partly due to the time taken for iNOS expression, but it also implies that once peroxynitrite is generated neuronal death is not immediate.

In both cases (LPS/IFN-γ/PMA or LPS/IFN-γ/BzATP), inhibitors of iNOS or NADPH oxidase or a scavenger of peroxynitrite prevented this neuronal death, implicating peroxynitrite as the potential mediator of neuronal death and the source of peroxynitrite as NO from iNOS and superoxide from NADPH oxidase. The putative peroxynitrite marker nitrotyrosine, was found in both neurons and some glia, implying that LPS/IFN-γ/PMA treatment does result in peroxynitrite production that reacts with neurons. Furthermore, the peroxynitrite decomposition catalyst prevents the occurrence of nitrotyrosine-positive neurons following LPS/IFN-γ/PMA treatment. FeTPPS has been shown to rapidly react and catalyse the decomposition of extracellular peroxynitrite [52] and inhibit tyrosine nitration [53]. The presence of nitrotyrosine immunoreactivity in glia did not appear to induce glial death. It has been found that glia can up-regulate their antioxidant defences to become more resistant to oxidative stress [54], which may explain the lack of change in glial morphology.

The mechanism of peroxynitrite-induced neuronal death is still unclear but has been proposed to involve DNA-damage induced PARP activation [55], damage to the mitochondrial respiratory chain [56], and lipid peroxidation [57]. It is still controversial whether peroxynitrite-induced neuronal death involves activation of the NMDA receptor [58,59]. We found that a blocker of the NMDA-receptor did not prevent the relatively acute neuronal death induced by LPS/IFN-γ/PMA or LPS/IFN-γ/BzATP, but did prevent the relatively slow neuronal death induced by LPS/IFN-γ/IL-1β or LPS/IFN-γ/AA, although in both cases death was prevented by a peroxynitrite decomposer. It is possible that low, sustained levels of peroxynitrite induce neuronal death via the NMDA receptor, whereas high, acute levels induce death by other means, but we have not directly tested this. We found that IL-1β or AA activated NADPH oxidase hydrogen peroxide production to a lesser extent than PMA but, like PMA, either IL-1β or AA synergised with LPS/IFN-γ to induce neuronal death mediated by peroxynitrite following activation of iNOS and NADPH oxidase. However the neuronal death induced by LPS/IFN-γ/IL-1β or LPS/IFN-γ/AA occurred over 6 days, rather than 2 days as with LPS/IFN-γ/PMA or LPS/IFN-γ/BzATP. This relative delay might be due to the lower level of NADPH oxidase activation and thus peroxynitrite production. Additionally, Il-1β or AA caused microglial proliferation during the 6-day cultures, which may have contributed to the delayed neuronal death. Recently we found that IL-1β or AA stimulated microglial proliferation in microglia-astrocyte cultures via hydrogen peroxide production from NADPH oxidase (manuscript in preparation). Here we have shown that IL-1β or AA stimulate the proliferation of microglia in neuronal-glial cultures, even in the presence of LPS/IFN-γ (which itself inhibits microglial proliferation). In order to test whether an increase in microglia would potentiate LPS/IFN-γ induced neuronal death, we added extra isolated microglia to the neuronal-glial culture, increasing the microglial population from 2% to 15% of cells in the co-culture. In such microglia-enriched cultures, LPS/IFN-γ induced neuronal death was greatly increased. These observations suggest that microglia are essential for inflammatory activated glia-induced neuronal death, and one reason for this may be the expression of NADPH oxidase, which is predominantly localised to microglia [24].

Transmissible spongiform encephalopathies (Prion diseases) are lethal neurodegenerative disorders characterised by the progressive accumulation of a protease resistant isoform (PrP^sc^) of the normal host prion protein (PrP^c^), in amyloid plaques [60]. An inflammatory response, predominantly mediated by microglia, is seen in post-mortem brain tissue, in transgenic models of the disease, and in culture [61]. A peptide, consisting of residues 106–126 (PrP106-126) of the human prion protein, replicates many of the pathological mechanisms involved in prion diseases and provides a good in vitro model. Contrary to published data [38,62], we observed no neurotoxicity following the addition of PrP106-126 alone to this mature neuronal-glial culture. We found that both prion protein and PrP106-126, but not a scrambled peptide, stimulated microglial proliferation when added to neuronal-glial cultures, and this proliferation was blocked by a NADPH oxidase inhibitor. Both prion protein and peptide were synergistic in killing neurons in the presence of glial iNOS, in a peroxynitrite and microglia-dependent mechanism. The addition of the cellular isoform of prion protein to cell cultures has previously been shown to have no toxic effects [34]. However, in the presence of glial iNOS we found it to induce significant levels of neuronal death, although significantly less than that induced by PrP106-126.

## Conclusion

We have shown that in a mature mixed culture of neurons and glia, activation of iNOS or NADPH oxidase alone does not result in substantial neuronal death, but that simultaneous activation of both is synergistic in killing co-cultured neurons. This neuronal death appears to be dependent on microglia, and microglial proliferation is itself stimulated by activating the NADPH oxidase. These results suggest a dual-key hypothesis for inflammatory neurodegeneration; i.e. that activation of glial iNOS or NADPH oxidase alone may be relatively benign, but when activated together they cause peroxynitrite-mediated neuronal death. The conditionality of NO/iNOS-induced neuronal death provides insight into the mechanisms of inflammatory neurodegeneration and suggests that microglial NADPH oxidase may be a key therapeutic target.

## Competing interests

The author(s) declare that they have no competing interests.

## Authors' contributions

PM participated in the design of this study, did the lab work, data analysis and wrote major parts of the paper. GCB conceived the study, participated in its design and helped to draft the manuscript. Both authors read and approved the final manuscript.
